# Representation of Time-Varying Stimuli by a Network Exhibiting Oscillations on a Faster Time Scale

**DOI:** 10.1371/journal.pcbi.1000370

**Published:** 2009-05-01

**Authors:** Maoz Shamir, Oded Ghitza, Steven Epstein, Nancy Kopell

**Affiliations:** 1 Center for BioDynamics, Boston University, Boston, Massachusetts, United States of America; 2 Department of Physiology, Ben-Gurion University of the Negev, Be'er-Sheva, Israel; Gatsby Computational Neuroscience Unit, United Kingdom

## Abstract

Sensory processing is associated with gamma frequency oscillations (30–80 Hz) in sensory cortices. This raises the question whether gamma oscillations can be directly involved in the representation of time-varying stimuli, including stimuli whose time scale is longer than a gamma cycle. We are interested in the ability of the system to reliably distinguish different stimuli while being robust to stimulus variations such as uniform time-warp. We address this issue with a dynamical model of spiking neurons and study the response to an asymmetric sawtooth input current over a range of shape parameters. These parameters describe how fast the input current rises and falls in time. Our network consists of inhibitory and excitatory populations that are sufficient for generating oscillations in the gamma range. The oscillations period is about one-third of the stimulus duration. Embedded in this network is a subpopulation of excitatory cells that respond to the sawtooth stimulus and a subpopulation of cells that respond to an onset cue. The intrinsic gamma oscillations generate a temporally sparse code for the external stimuli. In this code, an excitatory cell may fire a single spike during a gamma cycle, depending on its tuning properties and on the temporal structure of the specific input; the identity of the stimulus is coded by the list of excitatory cells that fire during each cycle. We quantify the properties of this representation in a series of simulations and show that the sparseness of the code makes it robust to uniform warping of the time scale. We find that resetting of the oscillation phase at stimulus onset is important for a reliable representation of the stimulus and that there is a tradeoff between the resolution of the neural representation of the stimulus and robustness to time-warp.

## Author Summary

Sensory processing of time-varying stimuli, such as speech, is associated with high-frequency oscillatory cortical activity, the functional significance of which is still unknown. One possibility is that the oscillations are part of a stimulus-encoding mechanism. Here, we investigate a computational model of such a mechanism, a spiking neuronal network whose intrinsic oscillations interact with external input (waveforms simulating short speech segments in a single acoustic frequency band) to encode stimuli that extend over a time interval longer than the oscillation's period. The network implements a temporally sparse encoding, whose robustness to time warping and neuronal noise we quantify. To our knowledge, this study is the first to demonstrate that a biophysically plausible model of oscillations occurring in the processing of auditory input may generate a representation of signals that span multiple oscillation cycles.

## Introduction

### General background

In recent years, there has been a growing interest in understanding how temporal information of sensory stimuli is encoded by sensory corticies (see, e.g., [1]–[8]). It has been shown that information about the features of the external stimulus is encoded in the fine temporal structure of the neural response (see, e.g., [8]–[15]). We are especially interested here in stimuli that have a natural hierarchy of temporal scales, such as speech and its components, including phones, diphones, words etc. Sensory processing has also been shown to be associated with the appearance of gamma oscillations in various sensory corticies (see, e.g., [16]–[20]). This raises the question whether the gamma oscillations can be directly involved in the representation of time-varying stimuli, including stimuli whose time scale is larger than that of a gamma cycle.

Such a model was suggested by Hopfield [5], and later was studied in the contex of diphone discrimination [21]. In this model subthreshold oscillatory input acts to coordinate the firing of cells so that a downstream neuron can read out a population code based on synchrony of firing. The implementation of this idea had a memory of about 200 ms, in a way that varied along a given stream of speech; the time scale of the memory depended on a dynamically changing “Lyapunov exponent”; the more negative this quantity, the shorter the memory and the more stable the representation. Thus, the longer memory was also associated with a less stable and less transparent representation. Here we build on the ideas in that paper about the synchronizing effects of gamma oscillations. However, to represent a signal having a natural time scale of more than one gamma period, we use multiple periods explicitly in the representation.

The aim of this paper is to show that this idea can be implemented robustly in the context of biophysically reasonable networks of neurons. The gamma oscillations are a product of the network, rather than an external input, and correspond to spiking events in the network, not subthreshold oscillations. We use a dynamical model of a network of spiking cells [22] that responds to a one-dimensional time-varying input in the shape of a sawtooth. Such a signal models the response of one cochlear frequency-band to a short speech stimulus, such as a diphone, that lasts several gamma cycles. We show that the oscillations produced by the network tend to discretize the neural response to the sawtooth. From this, we get a binary response of the population, based on which cells fire in which cycles. Using a simple measure of discriminability, we examine the reliability of the representation, and show that reliability requires an onset signal, something that is well known for sensory signals (see, e.g., [14],[23],[24],[25]). We also show that the representation is robust to moderate noise and time warp. In the Discussion, we compare the ideas of this paper with other work on coding (or recognition) of temporal patterns. We also discuss how hierarchies of oscillations in the nervous system may relate to the natural hierarchy of timescales in speech (phone, diphone, syllable, word, and sentence) and possible mechanisms for reading out the kind of code we suggest.

### Model stimulus

**Figure 1:**
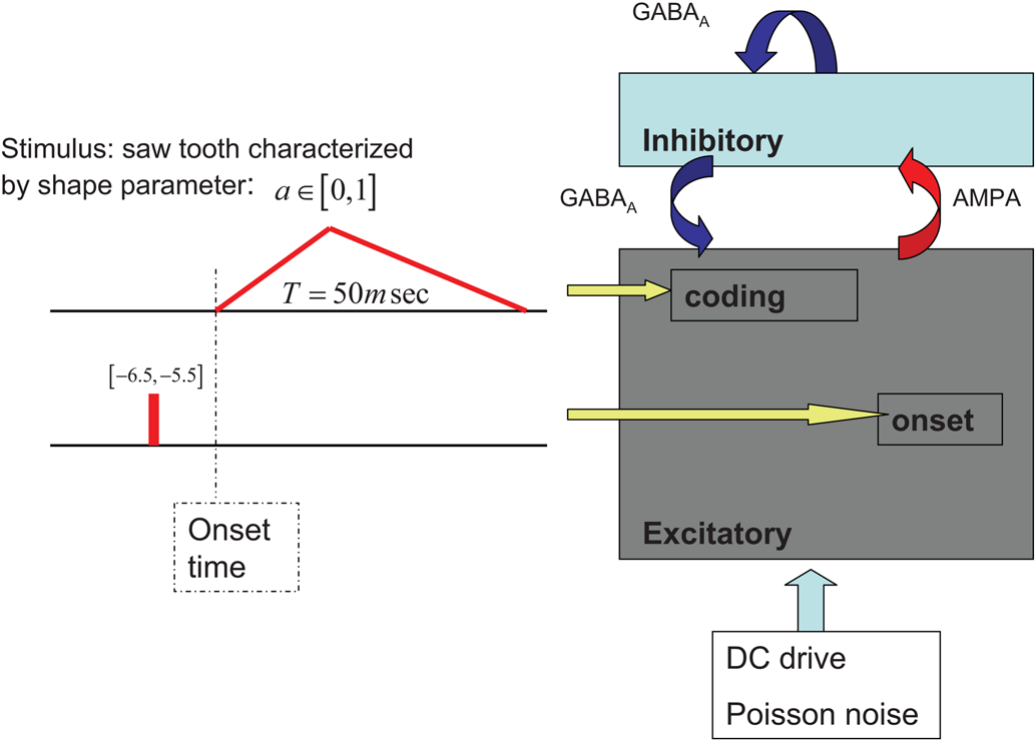
Network architecture. Neural population is composed of two large subpopulations: excitatory (E) and inhibitory (I). The E-to-I, I-to-E and I-to-I connectivity is all-to-all and are sufficient to generate and sustain oscillations in the gamma frequency range. Specifically, oscillation period was about 18 ms. Excitatory cells are further divided into three functional subpopulations according to their different inputs. The background subpopulation receives high DC current and is responsible for generating the intrinsic gamma oscillations. The onset subpopulation receives an onset signal and is responsible for resetting the oscillation phase to synchronize it with the stimulus onset. The last subpopulation is the coding population that receives the time dependent sawtooth input current.

Ultimately, we would like to study the representation of a diphone. A diphone is a speech segment, roughly from the middle of a phoneme to the middle of the phoneme following it. In a single cochlear frequency-band, the temporal fluctuations of the sound energy of a diphone can be represented in caricature by a single sawtooth waveform that mimics the dynamics of energy as it enters and leaves the frequency band. In this study we focus on the representation of sawtooth-shaped signals. Different sawtooths will be represented by a single shape parameter, 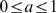, that specifies the time of the energy peak in the sawtooth from the beginning of the sawtooth, in units of the sawtooth period 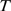 (see Figure 1). Unless otherwise stated we use a typical duration of 50 ms for the sawtooth stimulus, although we have tested the network response for slightly shorter and longer stimulus durations 40–100 ms. The advantage of using a simplistic abstract model for the input stimulus, instead of, for example, a real intensity profile taken from speech, is that it allows for systematic investigation of the representation which, in turn, facilitates the clear understanding of the properties of the representation.

### Model system: Response to sawtooth waveforms

The functional architecture of the network is depicted in Figure 1. The excitatory-inhibitory interactions are sufficient to generate and sustain oscillations in the gamma frequency range. Specifically, oscillation period was about 18 ms. Hence, the duration of the external stimulus (typically 50 ms) is about three network cycles. The oscillations are generated via a mechanism known as PING (Pyramidal-Interneuronal Network Gamma). Essentially, input from the excitatory cells cause the inhibitory population to fire and generate a volley of inhibition that synchronizes the network activity (see [22] for a fuller description).

Excitatory cells are further divided into three functional subpopulations according to their different inputs. The background subpopulation receives high DC current and is responsible for generating the intrinsic gamma oscillations. The onset subpopulation receives an onset signal and is responsible for resetting the oscillation phase to synchronize it with the stimulus onset. The last subpopulation is the coding population that receives the time dependent sawtooth input current. A more detailed description of the network and its dynamics appears in the Materials and Methods section below.

## Results

### Intrinsic oscillations discretize neural response

**Figure 2:**
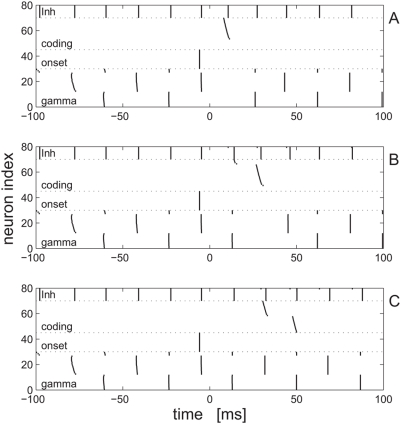
Network response to stimulus. Population response to three different stimulus shape parameters 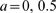 and 1 in A, B and C, respectively, are shown in a raster format. The x-axis is time. The stimulus is presented to the coding population at time 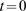 (onset signal is at *t* = −6.5 ms). Every line shows the spiking activity of a single cell in the population. The cells are ordered according to their functional subpopulation. At the bottom, lines 1–30, show spiking activity of cells in the excitatory background subpopulation. Lines 31–45 show the onset-response cells firing. Firing of cells in the coding population are plotted in lines 46–70. Cells in the coding population (cells 46–70) are plotted in an increasing order of their ‘sensitivity’ from bottom (cell 46 - least sensitive) to top (cell 70 - most sensitive). The spiking activity of cells in the inhibitory population appear in lines 71–80.

Figure 2 shows three examples of the population response to the external stimuli, in the absence of internal noise. The x-axis is time and every line shows the spiking events of a different cell in the population during the same trial. The cells are ordered according to their functional subpopulation. At the bottom (cells 1–30) is the excitatory background population that, together with the inhibitory population (top - cells 71–80), generate the intrinsic gamma oscillations. The onset-response population (cells 31–45) are responsible for resetting the phase of the intrinsic oscillations, thus, synchronizing them to the onset of the external stimulus. Cells in the coding population (25 cells, no. 46–70) are plotted in an increasing order of their ‘sensitivity’ from bottom (cell 46 - least sensitive) to top (cell 70 - most sensitive).

The three Figures 2A, 2B, and 2C show the population response to stimuli with three different shape parameter values 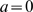, 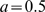 and 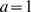, respectively. For a very fast-rising stimulus (Figure 2A, 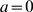), cells in the coding population will tend to fire in the first cycle immediately after the onset. For a slower-rising stimulus (Figure 2B, 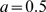), few cells will fire in the first cycle and most cells will fire in the second cycle after the onset. For a stimulus that rises even slower (Figure 2C, 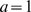), few cells will fire in the second cycle and most cells will fire in the third cycle after the onset.

Thus, intrinsic oscillations discretize the coding population response in the following sense: the external stimulus overlaps approximately three gamma cycles. Every cell can fire at most a single spike during every cycle. The specific spike pattern of every cell depends on its identity (i.e., different cells in the coding population have different sensitivity due to different DC input levels) as well as on the stimulus shape. Hence, the list of which cell fired during what cycle contains information about the stimulus shape. Below we define a binary representation of the neural response that will be used to quantify the information content of the response.

### Binary representation of population response

We represent the neural response by a binary matrix of size: [*number of coding cells*]×[*three gamma cycles*] (25×3 in our model). Matrix element (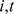) indicates whether cell 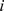 in the coding population fired (1) or did not fire (0) in the 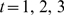 cycles following the stimulus onset. This choice of binary representation ignores information that may exist on a time scale finer than the gamma cycle.

**Figure 3:**
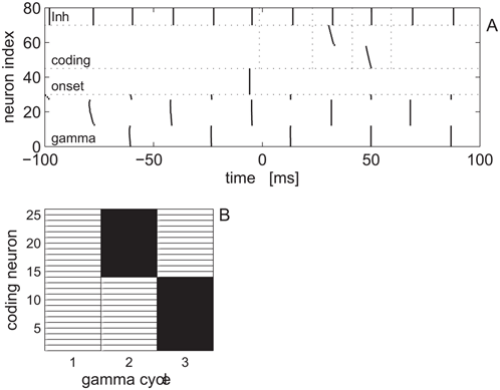
The binning procedure. A Population response to stimulus with shape parameter 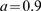 starting at time 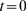 is shown in a raster format, similar to Figure 2. In our binary representation of the response, firing of cells in the coding population were binned to time intervals of single gamma cycles. The boundaries of the bins were defined by the mean spike times of the inhibitory cell population plus a 4.5 ms - shown by the vertical dotted lines. B Binary representation of the network response in A. The activity of every cell in the coding population during the three gamma cycles in which stimulus is presented is shown by a single row in the matrix. Every row is divided into three columns that show the firing of the cell during each gamma cycle in black (fired) and white (did not fire).

Figure 3 demonstrates the binning procedure (complete description of the procedure appears in Materials and Methods section, below). The mean firing time of the onset population (plus 4.5 ms) defines the start of the first bin. The boundaries of the bins are defined by the mean spike times of the inhibitory cell population plus 4.5 ms (vertical dotted lines in Figure 3A). Figure 3B shows the binary representation of the network response in Figure 3A. The activity of every cell in the coding population during the three gamma cycles in which stimulus is presented is shown by a single row. Every row is divided into three columns that show the firing of the cell during each cycle in black (fired) and white (did not fire).

### Quantifying the discriminability of population response

The information content can be quantified by measuring the discriminability of the binary representation of stimuli with different shapes. We chose a very simple readout mechanism, based on template matching. Every stimulus is associated with an internal binary template (see Materials and Methods). For a given response, the estimated sawtooth shape parameter is defined as the one associated with the closest template. Hamming distance was used as the distance measure between templates and input response. These choices were made due to their simplicity and the fact that they emphasize the binary nature of the neural responses. Neither the template nor the distance measure was chosen to optimize the estimation accuracy. We do not mean to suggest that the central nervous system uses this particular readout mechanism. Nevertheless, this readout is an appropriate metric for assessing the accuracy of population response in representing sawtooth-shape waveforms.

**Figure 4:**
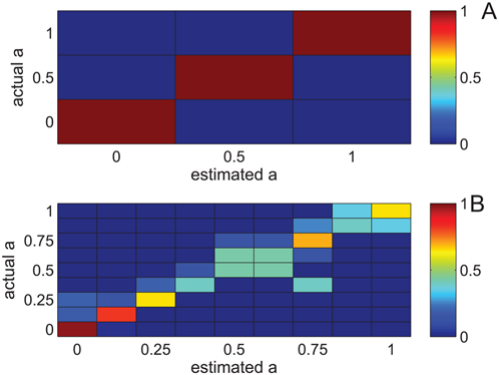
Confusion matrices in the absence of internal noise. The confusion matrix for discriminating A three alternatives: 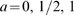 and B nine alternatives: 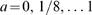 is shown in a color code. Element (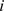, 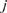) of the confusion matrix is defined as the conditional probability that the estimator takes the value 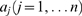, given the stimulus was 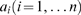. Every row of the confusion matrix was estimated by averaging over the different phase relations. Probability of correct classification is given by the mean of the diagonal of each confusion matrix is 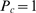 and 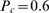 for the three and nine alternatives, respectively.

A convenient description of the readout discrimination power is the confusion matrix, 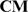 (see Materials and Methods). Figure 4 shows the confusion matrix for **A** three alternative shape parameter values: 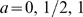 and **B** nine alternative shape parameter values: 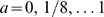. The probability of a correct classification provides a scalar summary of the of the confusion matrix. In the three alternative tasks, **A**, the system is always correct, the probability of correct classification is 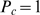 (chance level is 1/3). In the more difficult nine alternative task **B** performance decreases, 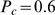 (chance level 1/9). However, errors in estimating the shape parameter, 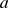, have a magnitude: 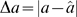 (where 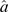 is the estimated shape parameter; see Materials and Methods equation 7). As can be seen from the confusion matrix, although the error rate increases, the errors are small, typically 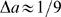 (the first off-diagonal elements in the confusion matrix).

**Figure 5:**
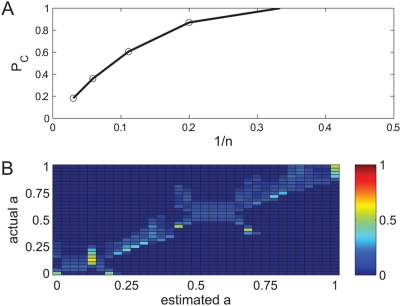
Discrimination at fine temporal resolution. A Effect of readout resolution on discrimination accuracy. The probability of correct discrimination 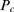 in the 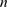 alternative forced choice is shown as a function of 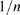. For each 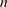, the probability of correct classification, 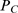, was estimated by averaging over the different phase relations. B Confusion matrix in the absence of internal noise for discriminating 33 alternatives: 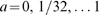. Every row of the confusion matrix was estimated by averaging over the different phase relations.

Figure 5A shows the the percent correct classification in an 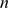 alternative (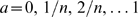) forced choice task, as a function of 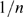. For large 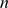, the percent correct decays to zero inversely with the number of alternatives, 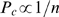. This results from a finite resolution in the representation of the shape parameter 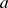. The confusion matrix in the case of 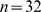 alternatives is shown in Figure 5B. As in Figure 4B, we observe that the confusion matrix has relatively large elements mainly close to the diagonal. Hence, although there is considerable probability of error, the magnitude of the error is typically small. This finite resolution can be quantified by the root mean square (RMS) of the estimation error, 
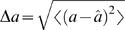
, where 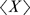 denotes average of 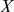 over different trials and phase relations. Here we obtain 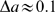. In order to obtain this resolution a reliable representation is required. Below we show the necessity of the phase resetting mechanism by the onset population for obtaining a reliable representation of the shape parameter.

### Reliable representation requires an onset signal

**Figure 6:**
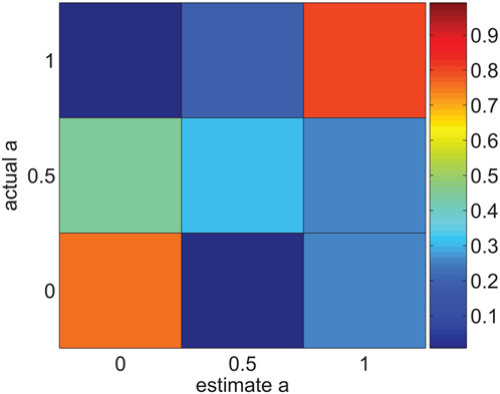
Confusion matrix without onset signal. The confusion matrix for discriminating three alternatives: 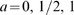 in the absence of an onset signal is shown in a color code. Every row of the confusion matrix was estimated by averaging over the different phase relations. It was estimated by averaging over the different phase relations. Probability of correct classification is 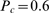, compare with 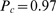 with onset signal (Figure 4A), chance level is 1/3.

Since network oscillations are intrinsic and the stimulus is external, the oscillation phase at the time of stimulus onset is arbitrary. In the absence of a phase resetting (synchronizing) mechanism, the same stimulus may elicit very different responses, depending on exact phase relation. This added variability of the neural responses to the stimulus increases the dispersion of the responses to the same stimulus around the template and can be thought of as added noise. Hence, the templates become less representative and the readout performance decreases. Figure 6 shows the confusion matrix in the three alternative task, 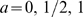, in the absence of the onset signal (see Figure 4A for comparison). As can be seen from the figure, the probability of correct classification decreased dramatically: 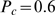, relative to 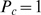, in the case with the onset signal. Nevertheless, performance is still above chance (chance level is 1/3).

It is important to note that the onset signal does not need to precede the stimulus. The requirement is that the onset signal activates the onset population before the coding population responds to the stimulus. In a diphone, typically, onset is shared among all frequency bands; hence, it provides a clear and robust signal. In a recent work Chase and Young [25] have demonstrated how an onset signal can be accurately reconstructed from the response of a population of inferior colliculus cells of the cat and then used to estimate the external stimulus.

Thus the onset response assists in stabilizing a reliable representation of the stimulus shape by the neural responses. However, it does not erase all traces of the past. Even with the presence of the onset signal, the neural response to the stimulus depends on the phase relation, but to a smaller extent. This variability in the neural responses to the same stimulus is, in part, responsible for the finite resolution of the representation 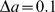 in the absence of intrinsic noise. Yet another factor that limits the resolution with which the network can represent the stimulus shape is our choice of binary representation. For example, one may imagine two close but different stimuli which elicit neural responses that differ by their exact spike times but fire during the same gamma cycle; these will be indistinguishable in our binary representation. Below we show that this insensitivity to exact spike timing is advantageous in representing time-warped stimuli.

### The representation is robust to moderate time-warp perturbations

**Figure 7:**
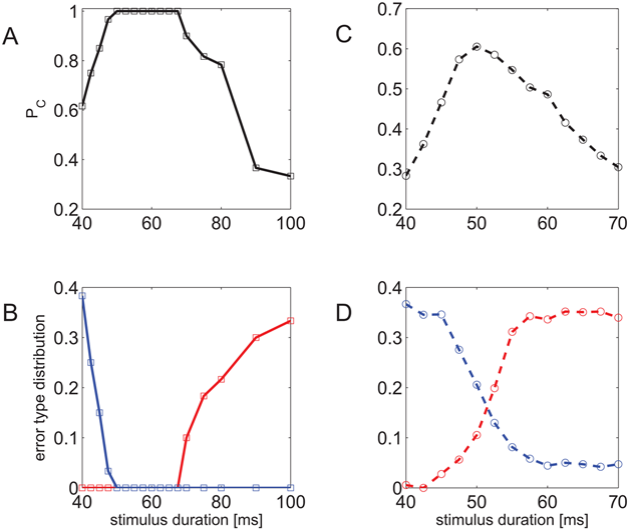
Robustness to time warping. A,C Probability of correct classification as a function of stimulus duration is shown for the three and nine alternative forced choice tasks in A and C, respectively. All network parameters remained unchanged. The templates were obtained from the network response to 50 ms stimulus duration. B,D Error type distribution for the three and nine alternative forced choice task in B and D, respectively. Probability of immediate up (down) error is shown in red (blue). Parameters used for the simulations in B,D are the same as in A,C respectively.

Time warp is a very common perturbation in speech signal. A desired property of speech representation is robustness to such perturbations. In order to study the robustness of our representation we modified the stimulus duration and measured our readout performance, keeping the same templates. Figure 7A shows the quality of representation, in terms of percent correct classification in the three alternative task, as a function of the stimulus duration. All network parameters remained unchanged. The templates were obtained from the network response to 50 ms stimulus duration, as in previous sections. As can be seen from the figure, probability of correct discrimination is maximal when the stimulus duration is 50 ms and decreases as the stimulus duration is changed. Nevertheless, there exists a large range of durations 45–75 ms in which probability of correct discrimination is well above chance level.

The type of errors caused by time warping of the stimulus depends on the specific time stretch. To see this, it is convenient to further classify errors into three groups: immediate-up, immediate-down and other. In the 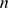 alternative forced choice task, errors in which stimulus 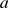 was estimated to be 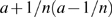 were classified as immediate-up (down). Figure 7B shows the error type distribution as a function of stimulus duration. As in Figure 7A, all network parameters remained unchanged and the templates were obtained from the network response to 50 ms stimulus duration. From the figure, one can see that immediate-down error rate (blue) increases when the stimulus duration is increased, whereas immediate-up error rate (red) increases when stimulus duration is decreased in the 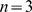 alternative forced choice task. Thus, error type follows the direction of time warping.

Figures 7C and 7D show the percent correct and error type distribution as in Figures 7A and 7B, respectively, in the 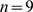 alternative forced choice task. Results in the 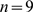 case are similar to the 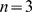. Probability of correct discrimination, 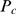, peaks at the duration used to obtain the templates, 50 ms, as the stimulus duration is changed, 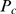 decreases. The immediate-down error rate is increased when stimulus duration is increased and vice versa for immediate-up error rate. Similarly, there exists a range of stimulus durations (of about 45–65 ms) for which probability of correct classification is well above chance level. However, this range is smaller for the 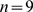 case than it is for the 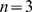 case. This difference is discussed below.

### Tradeoff: Resolution of representation and robustness to time-warp

**Figure 8:**
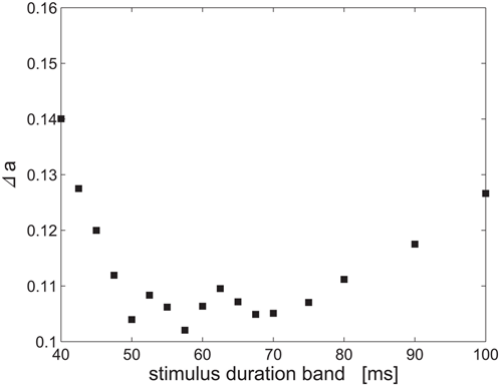
Tradeoff: sensitivity verses robustness to time warp. The RMS error of estimating the shape parameter in the 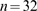 alternative forced choice is shown as a function of the band of stimuli durations.

Robustness to time warp comes at the expense of the resolution of the representation. This can be seen by comparing Figures 7A and 7B. When a higher resolution (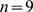 alternatives) is required, the range of durations in which the readout is robust to time warp is decreased, relative to the lower resolution case (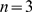 alternatives), see above. This notion can be further quantified by studying the RMS estimation error as a function of the amount of time warp of the stimulus. Figure 8 shows the RMS estimation error, 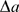, as a function of the amount of time warp of the stimulus duration. As can be seen from the figure, for stimulus durations of 50–70 ms the resolution fluctuates around its maximum (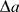 is minimal). The resolution decreases (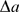 increases) as the amount of time warp increases in its magnitude, both above 70 ms and below 50 ms.

### The representation is robust to moderate intrinsic noise levels

**Figure 9:**
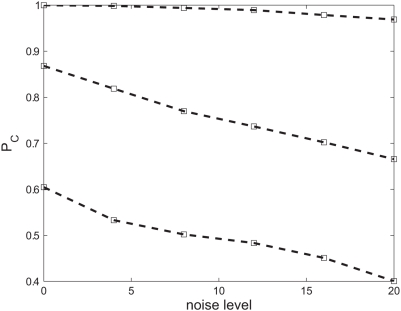
Probability of correct classification as function of the noise level for 3, 5 and 9 alternative forced choice, top to bottom. Noise level is shown as the independent random Poisson noise mean rate (per second) added to every cell's input. For every stimulus and every noise level neural responses were simulated for 20 different onset times and for every onset time for 10 different noise realizations. Neural responses were then divided, half for the training set to define the templates and half to test the generalization error. Results were further averaged over 100 divisions of training and generalization sets.

All of the above numerical simulations quantifying the network ability to represent time varying stimuli were done in a deterministic model, in the absence of intrinsic noise to the neural dynamics. For example, every inhibitory cell fired during every gamma cycle and every excitatory cell in the gamma generating population fired every other cycle. In a more realistic model [22],[26],[27] firing will be sparse and noisy, with oscillations that appear only on the network level. Thus, one should think of every cell in our deterministic model as an “effective cell”, representing the firing of a group of sparsely firing neurons. However, intrinsic noise that may cause spike time jitter, addition or deletion of spikes can have drastic detrimental effect on the quality of a temporal code [28],[29]. It is therefore important to test the sensitivity of this representation to intrinsic noise. Figure 9 shows the percent correct classification as a function of the input noise level for three, five and nine alternatives (top to bottom). As expected, the probability of correct discrimination is a monotonically decreasing function of noise level. Nevertheless, good performance levels are retained for moderate noise levels. Note, 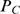 for three alternatives decreased by less than 5%, 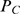 for five alternatives decreased by 23% and for nine alternatives decreased by 33%. This corresponds to a natural tradeoff of the representation resolution and robustness to intrinsic noise fluctuations.

## Discussion

Oscillations in the brain have been suggested to play a central role in various cognitive tasks, including attention [17],[18], navigation [30], memory [31] and motor planning [32]. In the context of speech processing, oscillations appear naturally, as almost all models of speech processing use oscillations, a clock signal or a pacemaker either explicitly or implicitly to take advantage of the natural hierarchy of timescales in the speech signal. Empirical findings suggest that oscillations in the auditory system may play an important role in spoken-language comprehension [20],[33]. The gamma frequency range (40–90 Hz), in particular, is widely found in the context of sensory processing [16]–[20].

The aim of this paper is to explore the use of oscillations in creating a representation of a time varying signal whose length is longer than the oscillation period. Using a family of signals, each in the shape of a sawtooth, but with different slopes, we have constructed a code using several gamma oscillations, with a total time interval about that of the signal. The gamma oscillations discretize the firing of a population of neurons, leading to a 3-bit binary representation. The representation of the shape parameter consists of a list of which cells in the coding population fired during what gamma cycle.

Typically, cells will fire at most once during the entire presentation of the stimulus. Hence, stimulus identity can be estimated by measuring the time interval between the firing of the onset cells and the firing of the coding cells. Every cell in the coding population is characterized by its sensitivity to the external stimulus, e.g., in Figure 2 cells 46–70 in the coding population are arranged in increasing order of sensitivity. This sensitivity dictates the firing order of cells in the coding population. Thus, the neural representation of the shape parameter is not arbitrary, but consists of natural firing order.

Our representation is sensitive to spike times with a resolution of a single gamma cycle (Figure 3). This finite temporal resolution limits the sensitivity with which temporal aspects of external stimuli can be coded (Figure 4). On the other hand, it provides robustness to fluctuations that affect the exact spike times. Those fluctuations include: stimulus variability, e.g., time warping (Figure 7), as well as intrinsic noise (Figure 9). There exists a natural tradeoff between the resolution of the representation and the robustness to fluctuations (Figure 8).

### Generality of our findings

In our numerical simulations we made certain choices that are required to define the system but are not essential for our qualitative results. We chose to represent the external stimulus by neural responses that extend over 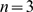 internal gamma cycles. The specific choice of 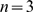 gamma cycles is arbitrary and our approach could be easily generalized to 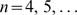 cycles. Larger 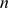 values imply that the stimulus can be represented to a finer resolution. However, finer resolution comes at the expense of robustness to noise and time-warping perturbations. The neurons in our simulations follow Hodgkin-Huxley dynamics (see Materials and Methods below). This choice is also not essential to our main conclusions. Other choices for the neural dynamics, such as integrate and fire, may generate representations that are different in their fine details but still preserve the central qualitative features reported here. Namely: the oscillations discretize the output, forming a binary representation that is robust to moderate levels of noise and time warping perturbations of the external stimulus and is characterized by a tradeoff of sensitivity and robustness. The essential features of our network are the architecture of a PING mechanism for generating the gamma oscillations and the manner in which the external stimulus interacts with the internal oscillations.

### Speech and hierarchy of nested oscillations

Speech is an important example of a time-varying signal. There is a natural hierarchy of timescales in speech: phone, diphone, syllable, word, and sentence. The time duration of phones and diphones is on the order of a few gamma cycles, while the duration of a word is roughly that of a theta cycles. Oscillations on different timescales in the auditory cortex have been shown to be organized hierarchically: delta modulates theta, theta modulates gamma [34]. These data support a view of a network with nested oscillations on different timescales [35]–[39]. Though a diphone can be correlated with a beta frequency period or multiple gamma frequency periods, we chose to explore the role of gamma frequency oscillations, since gamma oscillations are known to be prominent in early sensory processing (see, e.g., [16]–[20]), and to help produce cell assemblies [40].

The nesting of oscillations has a potential relationship to robustness to time warping. Empirical studies of speech [41],[42] as well as of birdsong [43] have shown positive correlations in time warping fluctuations of short speech and birdsong segments. For example, the degree of time warping of a specific syllable in Zebra finch song can be predicted, to a large extent, by the degree of time warping of previous syllables. Similarly, in speech, time warping fluctuations of nearby short speech segments are correlated. The correlated time stretch can be predicated by estimating a ‘tempo variable’, such as the prosody, that varies on a longer timescale. Such a tempo variable can be used by an oscillatory network to modulate its oscillation frequency to compensate for the time warp of the stimulus. The mechanism that we suggest for the time encoding lends itself naturally to such a tempo variable, since the PING gamma has increasing frequency with increased drive; any mechanism that can increase drive with faster prosody will produce more robustness to time warp variability of the auditory stimulus. The frequency of a slower but correlated rhythm, such as theta [44], could act as such a tempo variable. We note that theta rhythms and gamma rhythms sometimes covary in their frequencies [45]. The beta frequency may be associated with the onset signals.

### Relation to models of spoken-word recognition and other temporal patterns

In mainstream models of spoken-word recognition the speech waveform is processed by a front-end, providing a representation from which a phonetic transcription is generated. The sequence of phones recognized is then integrated into a form that results in a ‘pointer’ to a specific item in the lexicon. Phonetic transcription is usually accomplished by a search within a vocabulary of acoustic models of the phones. These models are statistical in nature, and the probabilistic model is acquired by training [46],[47]. While such Hidden Markov Models (HMMs) have shown themselves to be highly effective, it is reasonable to question certain properties of their basic structure as a model for biological systems of speech processing. The conditional independence assumption imposed by HMMs is a poor model for the dynamics in the speech signal [48]. It is also extremely difficult to model long-range dependencies with an HMM [49]. Thus, methods which can better model temporal-spectral dynamics inherent in speech are highly desirable.

Our long-term goal is to use the physiological aspects of speech processing to improve our understanding of speech representation. In the work discussed here, a first step in this endeavor, we quantify how our model represents a cartoon signal mimicking the response of one cochlear frequency-band to speech input. Many difficult questions have to be answered before we can implement this model as a front-end to a speech recognition system. For example, what is the discrimination power of the model for more realistic signals at the input of a single cochlear channel, e.g., for a set of signals that are different in shape, in duration, in amplitude? Can our model provide a stable representation with respect to time scale variations that conform with realistic phonemic variation (usually not a uniform time warp in nature)? How to synchronize an onset signal with the signals across several cochlear channels (with relative time alignment dictated by the speech source)? How to integrate across all cochlear channels? A system based on the principles of neuronal processing that answers these questions also has the potential to create a paradigm shift in the way that speech is processed by machines.

A closely related model was suggested by Hopfield [5]. The focus of this model was on readout of the activity of multiple integrate-and-fire neurons, each of which integrates over time the time-varying signal for a single “channel”. From the perspective of representing speech, the Hopfield model is complete; it suggests an architecture, with a subthreshold gamma oscillator at the core, in which all frequency bands are integrated via a well defined readout mechanism. Although we do not have a complete system yet, a comparison can be made between our model and Hopfield's for a single frequency-band signal. Hopfield used subthreshold oscillations to synchronize the firing across channels, forcing the cells to fire in a “window of opportunity”. Though the equations that embody the model have some memory beyond one cycle, the memory corresponds to a small negative Lyapunov exponent, which is also associated with lack of robustness. Thus, it is unclear how well this performs for a longer time-varying signal. In contrast, our model is not focused on readout, but on representation. The oscillations are used to discretize the signal across several periods, rather than to synchronize many channels. Spike times are determined by both the endogenous gamma rhythm and the external input. This mechanism allows the external stimulus to modulate the frequency of the intrinsic oscillation, unlike the fixed period in the Hopfield model.

The idea that a stimulus may be coded by a sequence of firings in discrete epochs has been discussed in the context of olfaction by Bazehnov et al. [50],[51]. There are two central differences between their work and ours: First, the Bazhenov et al. papers deal with a set of signals that all have the same temporal properties: they have a rise time of 100 ms and a decay time of 200 ms, unlike the sawtooth signals of the current work. Second, in [50],[51], the different signals excite different (possibly overlapping) sets of cells in the coding population, unlike the signals in the current paper, which all excite the same set of cells, but have different effects on them. Thus, the information in the signals is different from that of the Bazhenov papers and the coding strategy is different, even though both result in discretization. The differences in strategy are appropriate for the differences in the kinds of signals to be encoded: the energy in a given auditory frequency band has a varying temporal structure across the set of signals, for which a sawtooth of different shapes provides a characterization. There is no such structure in olfactory signals.

### Possible readout mechanisms

In the current work we did not simulate a neural network implementation of our readout mechanism. How can our readout be implemented? The approach taken by Hopfield lends itself to a simple readout mechanism based on simultaneity. Since our code has more than one “bit”, a more complex readout mechanism is necessary. There are many suggestions in the literature that might be modified to work for this example [14],[52].

Stimulus identity, in our model, can be estimated by measuring the *time* from the firing of the onset cells to the firing of the coding cell. This could be achieved, for example, by an integrator that starts integrating time at the onset response and stops integration at the response of the coding population neurons. Thus, a class of potential readout mechanisms is that of neuronal integrators. Of particular interest is a single cell integrator model of Loewenstein et al. [53] based on slow calcium dynamics in a dendrite of a single cell. In their model [53], calcium level along the dendrite transitions from high to low and the location of the transition point along the dendrite is determined by integration over time of dendritic inputs. Thus, the firing rate of the cell corresponds to the time integral of the cells' dendritic inputs. Readout of a multiple-bit code might make use of input to multiple dendritic branches. Other ways to estimate such times use long-term potentiation and depression [54] and physiological slow conductances [55]. The above are more appropriate to the current model than the Tempotron [56], which can distinguish arbitrary time varying inputs, but is unable to discriminate well temporal features that extend beyond its integration time.

### Directions for future work

In this work we studied a very simplified stimulus model. The envelope amplitude of a diphone stimulus in a single frequency channel was approximated by a sawtooth. Incorporating a wider range of envelope repertoire as well as ranges of amplitude and several frequency bands will result in a much richer temporal code and will, most likely, require a larger neural population. However, this richness of detail may impair the clarity of our results. Moreover, meaningful theoretical investigation along these lines requires a better empirical understanding of cortical oscillations during speech perception to yield the essential constraints for theory. For example, when studying a model of several frequency channels we must choose whether or not the onset stimulus and the oscillations are shared among the different channels. Different choices may lead to different results, without reason to choose one over another. The question of whether oscillations are shared is an empirical question. To pursue in a meaningful manner the theoretical framework begun in the current work requires empirical effort to characterize the interaction of neural oscillations with time varying stimuli across several frequency channels. The current framework motivates such empirical work by suggesting ways in which an external stimulus can interact with the dynamics that encodes the signal.

## Materials and Methods

### The model system

#### Model neurons

The neural model for the excitatory (E-cells) and the inhibitory (I-cells), as well as the gamma-generating mechanism (see below), used in this study, have been adopted from the work of Börgers, Epstein and Kopell [22]. Börgers et al. have used the neuronal model of Ermentrout and Kopell [57], which is a one-compartment reduction of the Traub and Miles [58] model. The basic structure of the model is the same for both E- and I-cells. In the absence of synaptic currents, the equations governing the membrane potential V takes the form of the classical Hodgkin-Huxley equation:
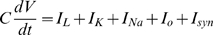
(1)

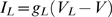
(2)

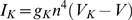
(3)

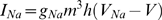
(4)The terms 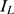, 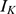 and 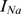 are standard leak, Potassium and Sodium currents, respectively. Following Börgers et al. (2005) we have used, 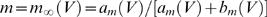 with 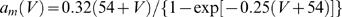 and *b_m_*(*V*) = 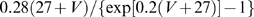, 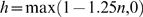, and the equation for 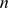 is 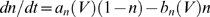 with 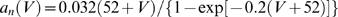 and 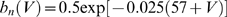. The letters 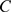, 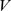, 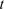, 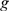, and 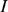 denote capacitance density, voltage, time, conductance density, and current density, respectively. The units used for these quantities are F/cm2, mV, ms, mS/cm^2^, and A/cm^2^, respectively. For brevity, units will often be omitted from here on. The parameter values of the model are 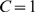, 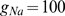, 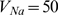, 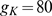, 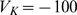, 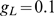, and 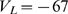. The term 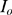 represents the baseline DC input current and the stimulus dependent time-varying current discussed below. The synaptic input to the cell, 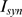, is discussed below.

In addition to the above mentioned currents, we have incorporated an M current in the E-cells, by adding the term
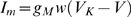
(5)to the right-hand side of Eq. (1), with 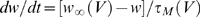, 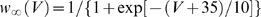, and 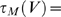
400/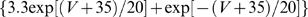. For the gamma and onset populations we have used 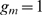, for the coding population we 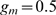 was used.

#### Model synapses

We model the excitatory synaptic connections to be mediated by AMPA (*α*-amino-3-hydroxy-5-methyl-4-isoxazolepropionic acid) receptors (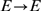 and 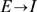), and the inhibitory by 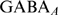 receptors (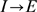 and 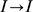). 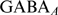 synapses are modeled by a term of the form 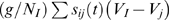 on the right-hand side of the equation governing the membrane potential of cell 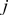, where 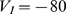, 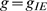 if cell 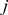 is excitatory, 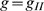 if cell 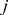 is inhibitory, and the sum extends over the indices 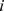 of the I-cells. The gating variables 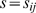 satisfy
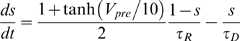
(6)with 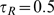, 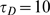, and 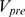 equal to the membrane potential of the presynaptic (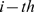) cell. Similarly, AMPA synapses are modeled by a term of the form 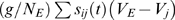 on the right-hand side of the equation governing the membrane potential of cell 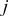, where 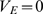, 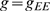 if cell 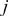 is excitatory, 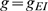 if cell 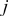 is inhibitory, and the sum extends over the indices 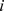 of the E-cells. The 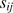 satisfy Eq. (6), with 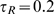 and 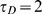.

#### Model networks

We consider networks of 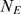 E-cells and 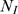 I-cells. The network architecture is depicted in Figure 1. In all simulation results, shown in this article, we have used 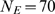 E-cells and 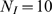 I-cells. Synaptic connectivity is all-to-all with 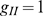, 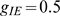, 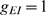 and 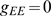. Note that for simplicity we omitted the 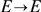 interactions. Note that the synaptic strengths are scaled by 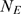 and 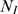, as described above in ‘Model synapse’.

The population of E-cells is further divided into three subpopulations according to their functional role. The functional role of a cell is determined by its inputs, see Figure 1. Each cell in the *gamma-generating* subpopulation (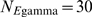) receives a strong baseline current input, 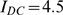, that is constant in time. Each *onset* cell (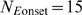) receives a constant input current input, 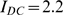, in addition to the onset signal, see ‘Model external stimulus’ below. Cells in the *coding* population (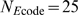) receive an array of constant currents ranging from maximal value of 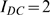, for the most sensitive cell down by steps of 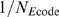, in addition to the external stimulus, see below.

*Noise:* In some of our simulations, each cell receives an independent Poisson stream of excitatory postsynaptic potentials (EPSPs) with a mean frequency of 0–20 Hz, see Figure 9. The associated synaptic conductance jumps to a maximal value instantaneously when the presynaptic spike arrives, then decays exponentially, with a time constant of 2 ms. The rate of the Poisson noise, 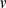, is constant across cells and in time and serves as a parameter that characterizes the noise strength.

#### Model external stimulus

The external stimulus input to the coding population is modeled by a sawtooth with a peak current of 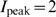 and duration time of 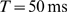. This represents in a simplified manner the increase and decrease of energy in a single frequency band during the pronunciation of a diphone. The sawtooth is further characterized by a single parameter, 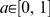 that measures the time of peak location from the beginning of the sawtooth, in units of the sawtooth period 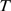. We shall term this parameter the asymmetry of the sawtooth hereafter. Different diphones are modeled by different values of 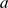.

The onset signal is modeled by a short rectangular current of amplitude 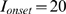 and duration of 1 ms that precedes the initial rise of the sawtooth by 6.5 ms, see Figure 1.

#### The readout mechanism

The readout used throughout this paper is based on template matching. Every stimulus was associated with a binary template of the network response, as in Figure 3. For a given response, the estimated shape parameter, 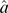, was determined by minimizing the distance between the response and the different templates
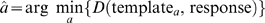
(7)when minimum is not unique, the estimator, 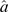, is chosen from the different minima randomly with equal probabilities. The templates were chosen in the following way. First the population binary response to the stimulus was averaged. In the absence of intrinsic noise, response was averaged over different phase relations between the stimulus onset and the intrinsic oscillation, for every 1 ms. In the presence of noise, response was averaged over 100 trials with different noise realizations and different phases. Then the the averaged response was clipped to obtain a binary template. For the distance measure, 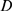, we used the Hamming distance. The Hamming distance was chosen for its simplicity. Neither the choice of template nor the choice of the distance measure were made to optimize the estimation accuracy.

#### The confusion matrix

Element (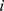, 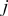) of the confusion matrix, **CM**, is defined as the conditional probability that the estimator takes the value 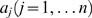, given the stimulus was 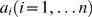. Every row of the confusion matrix was estimated by averaging over different phase relations in the absence of noise, or over 100 trials with different noise realizations and different phases in the case of intrinsic noise. Note that the probability of a correct classification, 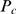, provides a scalar summary of the confusion matrix: 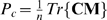, where 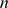 is the number of alternatives and *Tr*{**X**} denotes the trace of the matrix **X**.

#### Numerics

We solve the differential equations using the Matlab ode23 solver which implements the midpoint method with with adapting time-step. Initial conditions for neurons in the gamma-generating population are set to be uniformly spaced on their limit cycle in the absence of external stimulus. All other cells are initialized close to their resting point.
